# A new peak detection algorithm for MALDI mass spectrometry data based on a modified Asymmetric Pseudo-Voigt model

**DOI:** 10.1186/1471-2164-16-S12-S12

**Published:** 2015-12-09

**Authors:** Chalini D Wijetunge, Isaam Saeed, Berin A Boughton, Ute Roessner, Saman K Halgamuge

**Affiliations:** 1 Department of Mechanical Engineering, University of Melbourne, Parkville, VIC 3010, Australia; 2 Metabolomics Australia, School of Biosciences, University of Melbourne, Parkville, VIC 3010, Australia

**Keywords:** MALDI, Mass Spectrometry, Peak Detection

## Abstract

**Background:**

Mass Spectrometry (MS) is a ubiquitous analytical tool in biological research and is used to measure the mass-to-charge ratio of bio-molecules. Peak detection is the essential first step in MS data analysis. Precise estimation of peak parameters such as peak summit location and peak area are critical to identify underlying bio-molecules and to estimate their abundances accurately. We propose a new method to detect and quantify peaks in mass spectra. It uses dual-tree complex wavelet transformation along with Stein's unbiased risk estimator for spectra smoothing. Then, a new method, based on the modified Asymmetric Pseudo-Voigt (mAPV) model and hierarchical particle swarm optimization, is used for peak parameter estimation.

**Results:**

Using simulated data, we demonstrated the benefit of using the mAPV model over Gaussian, Lorentz and Bi-Gaussian functions for MS peak modelling. The proposed mAPV model achieved the best fitting accuracy for asymmetric peaks, with lower percentage errors in peak summit location estimation, which were 0.17% to 4.46% less than that of the other models. It also outperformed the other models in peak area estimation, delivering lower percentage errors, which were about 0.7% less than its closest competitor - the Bi-Gaussian model. In addition, using data generated from a MALDI-TOF computer model, we showed that the proposed overall algorithm outperformed the existing methods mainly in terms of sensitivity. It achieved a sensitivity of 85%, compared to 77% and 71% of the two benchmark algorithms, continuous wavelet transformation based method and Cromwell respectively.

**Conclusions:**

The proposed algorithm is particularly useful for peak detection and parameter estimation in MS data with overlapping peak distributions and asymmetric peaks. The algorithm is implemented using MATLAB and the source code is freely available at http://mapv.sourceforge.net.

## Background

Matrix Assisted Laser Desorption Ionization - Mass spectrometry (MALDI-MS) is a well-established analytical technique in biological research. In particular, it is being widely used in proteomics, metabolomics and lipidomics studies [1-4]. MALDI-MS can be used to measure amounts of bio-molecules in complex biological matrices thereby discovering differentially expressed bio-molecules. Over the past decade MALDI Imaging Mass Spectrometry (IMS) approaches, which measure the spatial distribution of bio-molecules in thin sections of tissue, have been rapidly developed [5-7]. MALDI-IMS relies upon collecting many MALDI-MS spectra in a two dimensional array. A typical dataset generated by these techniques may contain hundreds or thousands of spectra, each with hundreds to thousands of intensity measurements of peaks corresponding to various bio-molecules.

Detection of peaks in mass spectra is the initial step in MALDI-MS data analysis. Enormous care should be taken to perform this step as accurately as possible because the errors that occur in this step highly affect the performance of subsequent steps and can possibly lead to wrong conclusions. In general, the peak detection procedure consists of three main steps namely: 1) spectra smoothing, 2) baseline correction and 3) peak picking [8,9].

Even though the peaks corresponding to bio-molecules appear as local maxima in a spectrum, detecting these peaks is a challenge due to the high background noise. The background noise that is measured at the detector will cause a non-uniform background in the acquired spectra and the generation of confounding signals. These signals can suppress the important low-amplitude peaks corresponding to low-abundance bio-molecules in mass spectra. Moreover, they can cause a decreasing curve in the mass spectrum, which is known as baseline, making the peak detection process a challenge. Therefore, it is crucial to perform spectra de-noising and baseline correction prior to peak picking.

In the literature, various methods such as Gaussian filtering, Average filtering and several wavelet transformation based methods have been utilized in spectra de-noising [9]. Usually, in mass spectra, noise decreases along the spectrum and the peaks turn out to be shorter and wider at higher masses. Therefore, simple fixed window based methods like Average and Gaussian filters often fail to produce adequate results [10]. As an alternative, wavelet transformation based methods have also been used for spectra de-noising [10-12]. Basically there are two types of wavelet transformation based methods namely Discrete Wavelet Transformation (DWT) and Continuous Wavelet Transformation (CWT). These methods transform mass spectra into the wavelet domain and represent them in terms of wavelet coefficients in multiple scales. CWT computes wavelet transforms on every scale while capturing more information regarding the peaks in the mass spectrum. However, it is redundant and less efficient. On the other hand, DWT is non-redundant as it operates only on the required number of scales. However, it is shift-variant, meaning that a small shift in the starting position of the spectrum can cause a major drop in performance. In order to overcome this limitation, Coombes *et al*. proposed Undecimated Discrete Wavelet Transformation (UDWT), which is an improved shift-invariant version of DWT, for spectra de-noising [11].

After de-noising, baseline should be removed from each spectrum. Various methods based on monotone minimum, linear interpolation and moving average of minima have been utilized in baseline estimation [9,11]. However, if CWT is used for spectra smoothing, a separate step for baseline removal is not required as it has the ability to automatically remove the baseline [10].

After de-noising and baseline removal, peak picking becomes less challenging. Numerous peak finding methods have been proposed in the literature. Some studies define peaks as local maxima in a spectrum [2,11]. Signal to noise ratio of peaks has also been widely used for defining peaks [2,11,13]. Du *et al*. proposed a method based on ridge lines for peak picking after using CWT [10].

Precise estimation of peak parameters such as peak summit location and peak area is of high importance. Inaccurately estimated peak locations can cause problems when identifying underlying bio-molecules and can possibly lead to wrong predictions. Also, peak area is a better estimation for molecular abundance than the peak intensity [14,15]. However, the above mentioned peak picking methods provide less accurate estimations for peak parameters. Therefore, in order to estimate peak parameters more accurately, model-based peak picking methods have also been used. These methods use various model functions such as Gaussian and Lorentz to fit peaks [16,17]. These models produce inaccurate results when the peaks are asymmetric. In order to overcome this limitation, the Bi-Gaussian model has been proposed [18]. However, it tends to perform poorly when dealing with peaks that follow different shapes other than Bi-Gaussian. Recently, Bayesian non-parametric models have also been proposed for peak detection in MALDI Time of Flight (TOF) mass spectra [19]. As these models have been developed incorporating the properties restricted to the TOF analyser, unlike the other algorithms, they cannot be used for peak detection in other types of MALDI mass spectra. Also, the high computational cost of these methods hinders their practical application.

This paper proposes a new peak detection algorithm based on (i) dual-tree complex wavelet transformation and Stein's unbiased risk estimator for spectra smoothing; (ii) monotone local minimum curve fitting for baseline correction and (iii) the modified Asymmetric Pseudo-Voigt model together with hierarchical particle swarm optimization for peak modelling and parameter estimation.

## Methods

### Spectra smoothing

For spectra smoothing, we used an improved version of the conventional DWT method namely Dual-Tree Complex Wavelet Transformation (DT-CWT). The main advantage of DT-CWT over DWT is its ability to achieve near shift-invariance. Also, it is more efficient than Undecimated Discrete Wavelet Transformation (UDWT), which is another shift-invariant version of DWT, as the former has a relatively small redundancy factor. Hence it has shown promising results in different areas outperforming DWT and its other variants [20].

For DT-CWT, a q-shift Hilbert pair of wavelets was used. Mostly in the wavelet domain, true peaks are represented by relatively large wavelet coefficients and noise is represented by relatively small wavelet coefficients. Therefore, after transforming mass spectra to the wavelet domain, the coefficients below a certain threshold were set to zero in order to eliminate noise and then the resultant coefficients were transformed back into the intensity domain. This threshold value should be selected carefully because a lower threshold value leads to under-smoothing and a higher threshold value results in over-smoothing. In this study, Stein's Unbiased Risk Estimator (SURE) was used to calculate the threshold value for each level in the wavelet domain. SURE is an unbiased estimator that can be used to get an estimate of the risk or the mean-squared error for a threshold value [21]. Therefore, an optimal threshold value can be selected by minimizing the estimated risk. For detailed information about SURE, see Appendix 1.

We observed in most mass spectrometry data, that the noise decreases along the spectrum. Therefore, at the beginning, the raw spectrum was split into 4 equal sections and each section was smoothed separately using the DT-CWT method.

### Baseline correction

In general, the baseline in MALDI-MS data decreases at the beginning and then stays constant. Therefore, we estimated the baseline by fitting a monotone local minimum curve, which follows the spectrum when it is decreasing and remains unchanged when the spectrum is increasing, to the smoothed spectrum. For detailed information about this method, see Yang *et al*. [9]. The baseline estimated in this manner should be removed from the smoothed spectrum.

### Peak picking

After de-noising and baseline correction, the algorithm used for peak picking and peak parameter estimation in mass spectra can be summarized as follows:

1. Locate valleys (local minima) in the smoothed and baseline-corrected spectrum and split the spectrum into groups of data points at the valleys. Suppose the spectrum is split into *k *groups of data points.

2. For each group (*j *= 1,2,..., *k*) of data points in the spectrum,

   2.1 Fit a peak to the data points in component *j*, using the modified Asymmetric Pseudo-Voigt (mAPV) model.

        Let $\theta =\left(H,\sigma_{1},\sigma_{2},\beta_{1},\beta_{2},\alpha \right)$ be the parameter vector that needs to be optimized (these peak parameters are introduced in the next sub-section).

        Use the Hierarchical Particle Swarm Optimization (HPSO) method to obtain the optimized peak parameters ($\hat{\theta }$) based on equation (1):

(1) $$\hat{\theta }=\text{arg}\text{min }F\left(\theta \right).$$

        *F*(*θ*) is the objective function given by:

(2) $$F\left(\theta \right)=\underset{m}{\sum }{\left[V\left(m,\theta \right)-S(m)\right]}^{2},$$

        where *S*(*m*) is the actual intensity value at mass *m *and *V*(*m*, *θ*) represents the fitted value.

        (The mAPV model and the HPSO method are explained in detail in the next two sub-sections.)

   2.2 Calculate the area of the fitted peak and if it is smaller than a threshold value, then eliminate component *j *from further analysis.

   2.3 Check the peak width. If it is greater than a threshold value, there is a possibility that it contains multiple overlapping peaks. Therefore, locate the valleys between the starting and ending points and split component *j *into subgroups at the valleys. For each subgroup of data points, repeat steps 2.1, 2.2 and 2.5.

   2.4 Check the level of peak asymmetry. If *σ*_1 _and *σ*_2 _denote the standard deviations of the two halves of the peak, then peak asymmetry (*μ*) can be determined as follows:

(3) $$\mu =\frac{max\left(\sigma_{1},\sigma_{2}\right)}{min\left(\sigma_{1},\sigma_{2}\right)}.$$

        If the peak is highly asymmetric (*μ *> 2), there is a possibility that it contains multiple overlapping peaks. Such overlapping peaks need to be decomposed accurately. Therefore, if the peak is highly asymmetric and if it is possible to locate valley points between the starting and ending points, then split *j *into subgroups at the valleys. For each subgroup of data points, repeat steps 2.1, 2.2 and 2.5.

   2.5 Record optimized peak parameters$\left(\hat{\theta }=Ĥ,{\hat{\sigma }}_{1},{\hat{\sigma }}_{2},{\hat{\beta }}_{1},{\hat{\beta }}_{2},\hat{\alpha }\right)$.

### The modified Asymmetric Pseudo-Voigt (mAPV) peak model

**Figure 1:**
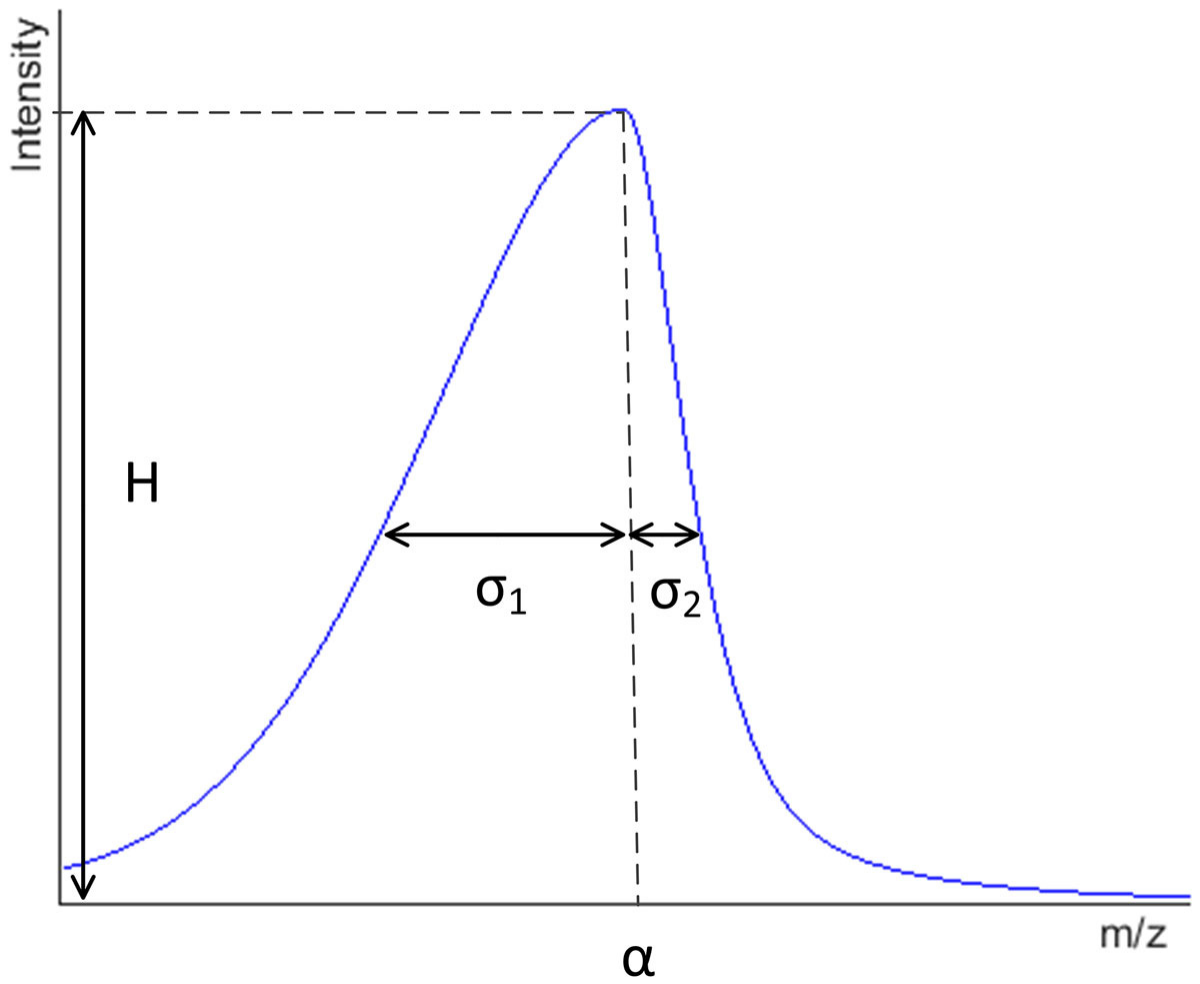
**The modified Asymmetric Pseudo-Voigt Function**. H, α, σ_1 _and σ_2 _represent the height of the peak, peak summit location, standard deviation of the first half of the peak, and that of the second half of the peak respectively.

We propose the mAPV model, which is a linear combination of Gaussian and Lorentz functions, to fit MALDI-MS peaks (Figure 1). It has 6 parameters namely height of the peak (*H*), location of the peak summit (*α*), the standard deviation of the first half of the peak (*σ*_1_), that of the second half of the peak (*σ*_2_), the fraction of Lorentz function used in the first half of the peak (*β*_1_) and that of the Lorentz function used in the second half of the peak (*β*_2_). When this model is used, the intensity values of a peak in a mass spectrum can be modelled as a function of mass values (*m*) according to equation (4):

(4) $$V\left(m\right)=\left\{\begin{array}{c} H\times \left\{\beta_{1}\left[\frac{1}{1+{\left(\frac{m-\alpha }{\sigma_{1}}\right)}^{2}}\right]+\left(1-\beta_{1}\right)e^{\left[-ln\left(2\right){\left(\frac{m-\alpha }{\sigma_{1}}\right)}^{2}\right]}\right\};m<\alpha \\ H\times \left\{\beta_{2}\left[\frac{1}{1+{\left(\frac{m-\alpha }{\sigma_{2}}\right)}^{2}}\right]+\left(1-\beta_{2}\right)e^{\left[-ln\left(2\right){\left(\frac{m-\alpha }{\sigma_{2}}\right)}^{2}\right]}\right\};m\geq \alpha \end{array}\right).$$

Both *β*_1 _and *β*_2 _lie in the range between 0 and 1. Hence, (1 - *β*_1_) and (1 - *β*_2_) represent the fractions of Gaussian function used in the first and second halves of the peak respectively.

The proposed mAPV model is a customization of the Asymmetric Pseudo-Voigt (APV) function used in the literature [22,23]. Since the standard APV function has only one β parameter, it cannot adequately model mass spectral peaks having dissimilar proportions of Lorentz and Gaussian functions in the two halves of the peak (m < α and m ≥ α). In contrast, the two β parameters allow the mAPV model to take dissimilar values for the proportions of Lorentz and Gaussian functions in the two halves of the peak.

### Hierarchical particle swarm optimization (HPSO)

Peak fitting using the mAPV model is basically an optimization problem. It is required to minimize the difference between the fitted values and the actual values. Hence, the objective function is defined as in equation (2).

We used the HPSO method proposed by Ratnaweera *et al*., which is an improved version of the conventional Particle Swarm Optimization (PSO) algorithm, to obtain the optimized peak parameters [24]. It is a population-based optimization technique which starts by randomly initializing the population of particles in the search space. Then, it finds the global best solution by adjusting the path of each particle, towards its own best location and the best particle of the entire swarm. At each time step, this best particle of the entire swarm is found according to a user defined objective function [24].

Let n be the number of particles in the swarm and *d *be the dimensionality of the search space. In this application, *d *equals to 6 since there are 6 peak parameters that need to be optimized. In the *d *dimensional search space, each particle *i *(1 ≤ *i *≤ n) has the following features: its position vector$X_{i}=\left(x_{i1},x_{i2},\ldots ,x_{id}\right)$, its velocity vector $V_{i}=\left(v_{i1},v_{i2},\ldots ,v_{id}\right)$ and its personal best position$P_{i}=\left(p_{i1},p_{i2},\ldots ,p_{id}\right)$. Let $P_{g}=\left(p_{g1},p_{g2},\ldots ,p_{gd}\right)$ be the best particle found so far. Then, this method updates the velocities and positions of the particles according to the following pseudocode:

$$\begin{array}{ccc} for(d=1\;to\;6) & & \\ \;v_{id} & = & c_{1}\times rand\left(.\right)\times \left(p_{id}-x_{id}\right)+c_{2}\times Rand\left(.\right)\times \left(p_{gd}-x_{id}\right)\\ x_{id} & = & x_{id}+v_{id}\\ \;end \end{array}$$

where *rand*(·) and *Rand*(·) are two random numbers in the range [0,1] that are separately generated using the uniform distribution. *c*_1 _and *c*_2 _are calculated according to equations (5) and (6) respectively:

(5) $$c_{1}=\left(c_{1f}-c_{1i}\right)\frac{iter}{MAXITR}+c_{1i},$$

(6) $$c_{2}=\left(c_{2f}-c_{2i}\right)\frac{iter}{MAXITR}+c_{2i},$$

where *iter *denotes the present iteration number, *MAXITR *denotes the maximum number of acceptable iterations and *c*_1*f*_, *c*_1*i*_, *c*_2*f *_and *c*_2*i *_are constants. In this study, we used the best values suggested by Ratnaweera *et al*. for these constants [24]. The complete pseudocode of the HPSO algorithm is available in Appendix 2.

### Dataset 1: Simulation data to evaluate the mAPV model

A comprehensive simulation study was conducted in order to assess the performance of the proposed mAPV peak model. It was compared with three other widely used peak models namely Gaussian, Lorentz and Bi-Gaussian (see Appendix 3). In order to generate data for this comparison study, an approach similar to Yu and Peng, where the data were simulated using a 3-component Bi-Gaussian mixture model was used [18]. In this study, the data were generated using a 2-component mAPV model. The 12 parameters used in this simulation study along with their values are listed in Additional file 1. By varying 7 out of those 12 parameters, 2700 parameter combinations were created in order to assess the peak fitting accuracy at different levels of peak asymmetry and peak overlap. In each parameter setting, the intensity values of each component (peak) were obtained from the mAPV function in equation (4). Then, the intensity values of both components were added together and noise was introduced in order to make the peak fitting process more challenging. In this study, each parameter setting was tested 100 times.

### Dataset 2: Simulation data generated from the MALDI-TOF computer model

It is difficult to evaluate the performance of the proposed overall algorithm using real MS data as the true peak parameters are usually not known. Therefore, in order to validate the competency of the proposed peak detection algorithm, we used a publicly available simulation dataset [12,15]. It consists of 25 groups of data each containing 100 spectra. This simulation dataset was created using a computer model that incorporates the physical properties of MALDI Time-of-Flight (TOF) MS [15]. Given a list of peaks with mass-to-charge (*m/z*) values and abundances, this computer model produces a virtual spectrum. Therefore, the true peak list corresponding to each generated spectrum is known. Coombes *et al*. showed that the spectra simulated from this model reflect the important characteristics of real MALDI-TOF-MS spectra [15].

## Results and discussion

### Performance assessment of the mAPV model against the other peak models

**Figure 2:**
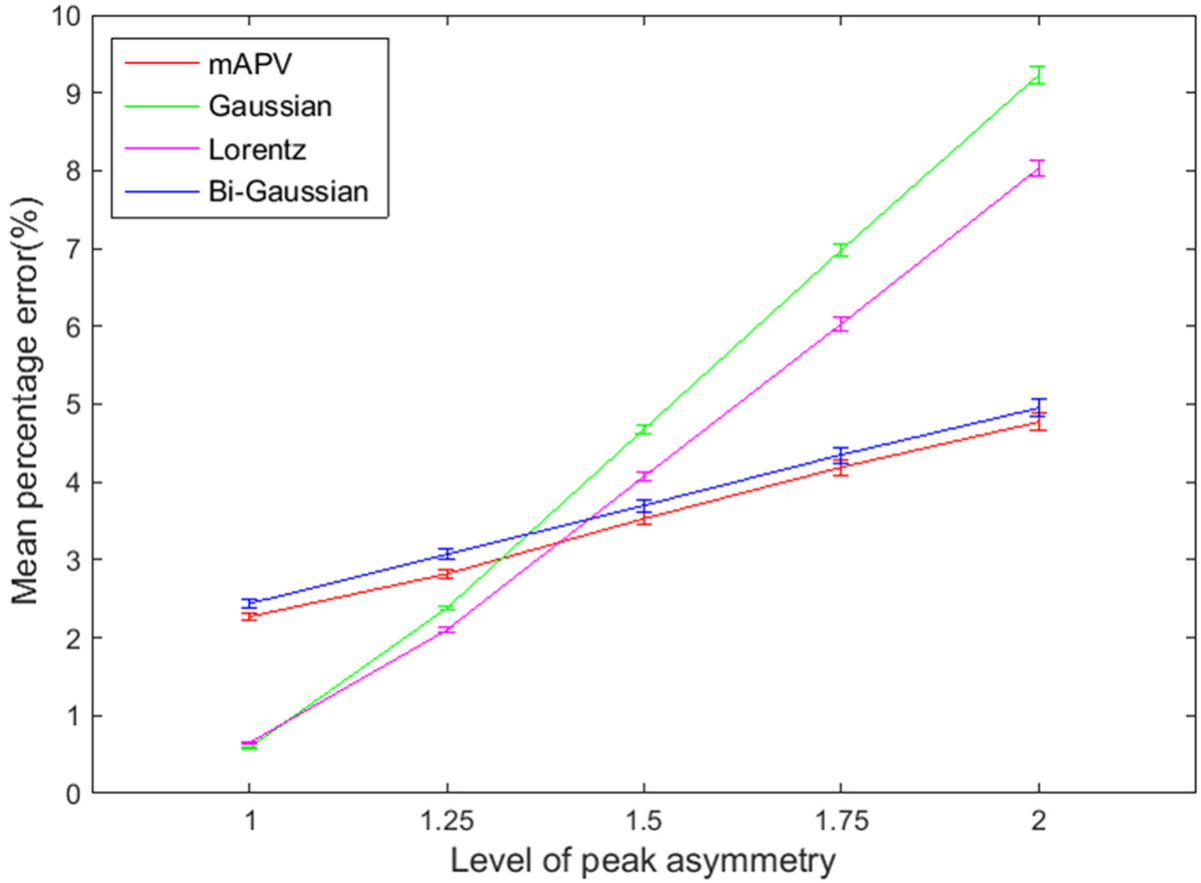
**Performance of the mAPV, Gaussian, Lorentz and Bi-Gaussian models in peak summit location estimation**. Mean percentage errors of the four models at different levels of peak asymmetry are shown.

Using the first simulation dataset, we compared the mean percentage error in estimating the peak summit location between the Gaussian, Lorentz, Bi-Gaussian and the mAPV models (Figure 2, Table 1). HPSO was used with all these models for peak fitting in order to make this comparison unbiased. The proposed mAPV model showed an advantage over the other peak models when the peaks were asymmetric, delivering lower mean percentage errors which were 0.17% to 4.46% less than that of the other models. Especially when the peaks were highly asymmetric (μ = 2), the benefit of the mAPV model was quite evident as it estimated the peak summit locations with a mean percentage error of 4.77%, in comparison with 9.23%, 8.03% and 4.95% of Gaussian, Lorentz and Bi-Gaussian models respectively. However, when the peaks were symmetric, Gaussian and Lorentz functions delivered less mean percentage errors than the mAPV model. The mean percentage error values delivered by the mAPV model were 0.16% to 0.25% less than that of the Bi-Gaussian model. In MS peak detection, this difference in peak summit location estimation is practically important as it could lead to the misidentification of an isotope or a completely different bio-molecule. In many cases, mass accuracy of more than two decimal places is essential for identifying the underlying bio-molecule accurately. For example, the exact mass of purine (C_5_H_4_N_4_) is 120.0436 mass units (u) and that of acetophenone (C_8_H_8_O) is 120.0575u. Hence, their exact mass difference is 0.0139u [25]. Moreover, two amino acids namely lysine (C_6_H_14_N_2_O_2_) and glutamine (C_5_H_10_N_2_O_3_) differ in exact mass by only 0.036u [26]. In both these cases, at least a mass accuracy of up to 2 decimal places is required, in order to allow unambiguous assignment of molecular formula which aids in identification.

**Table 1:** Performance of the mAPV model against the other peak models in peak summit location estimation.

Peak asymmetry (μ)	Mean percentage error (%)			
	
	Gaussian	Lorentz	Bi-Gaussian	mAPV
1	0.59 ± 0.01	0.65 ± 0.02	2.44 ± 0.05	2.27 ± 0.05
				
1.25	2.38 ± 0.03	2.10 ± 0.04	3.07 ± 0.07	2.82 ± 0.06
				
1.5	4.68 ± 0.06	4.07 ± 0.06	3.70 ± 0.08	3.53 ± 0.08
				
1.75	6.98 ± 0.08	6.03 ± 0.08	4.35 ± 0.10	4.19 ± 0.10
				
2	9.23 ± 0.11	8.03 ± 0.10	4.95 ± 0.11	4.77 ± 0.11

Values are expressed as mean ± standard error (SE).

Secondly, we compared the mean percentage error in peak area quantification between the four peak models (Table 2). Compared to the other peak models, the mAPV model yielded much smaller mean percentage errors of 2.47%, 2.33% and 2.26% for symmetric (μ = 1), moderately asymmetric (μ = 1.5) and highly asymmetric (μ = 2) peaks respectively. These mean percentage error values were about 0.7% less than that of its closest competitor **- **the Bi-Gaussian model. Therefore, when the peak area estimation was considered, the proposed peak model outperformed all the other models in all scenarios. Since the mAPV model can represent Gaussian, Lorentz and Bi-Gaussian models individually as well as various combinations of them, it is obvious that it outperforms or at least equally performing as one of the three methods.

**Table 2:** Performance of the mAPV model against the other peak models in peak area estimation.

Peak asymmetry (μ)	Mean percentage error (%)			
	
	Gaussian	Lorentz	Bi-Gaussian	mAPV
1	3.58 ± 0.07	4.44 ± 0.11	3.20 ± 0.05	2.47 ± 0.06
				
1.25	3.49 ± 0.06	4.21 ± 0.10	3.13 ± 0.05	2.36 ± 0.06
				
1.5	3.54 ± 0.07	3.95 ± 0.08	3.05 ± 0.05	2.33 ± 0.05
				
1.75	3.65 ± 0.07	3.53 ± 0.07	3.04 ± 0.06	2.30 ± 0.05
				
2	3.78 ± 0.08	3.13 ± 0.06	2.98 ± 0.06	2.26 ± 0.05

Values are expressed as mean ± SE.

**Figure 3:**
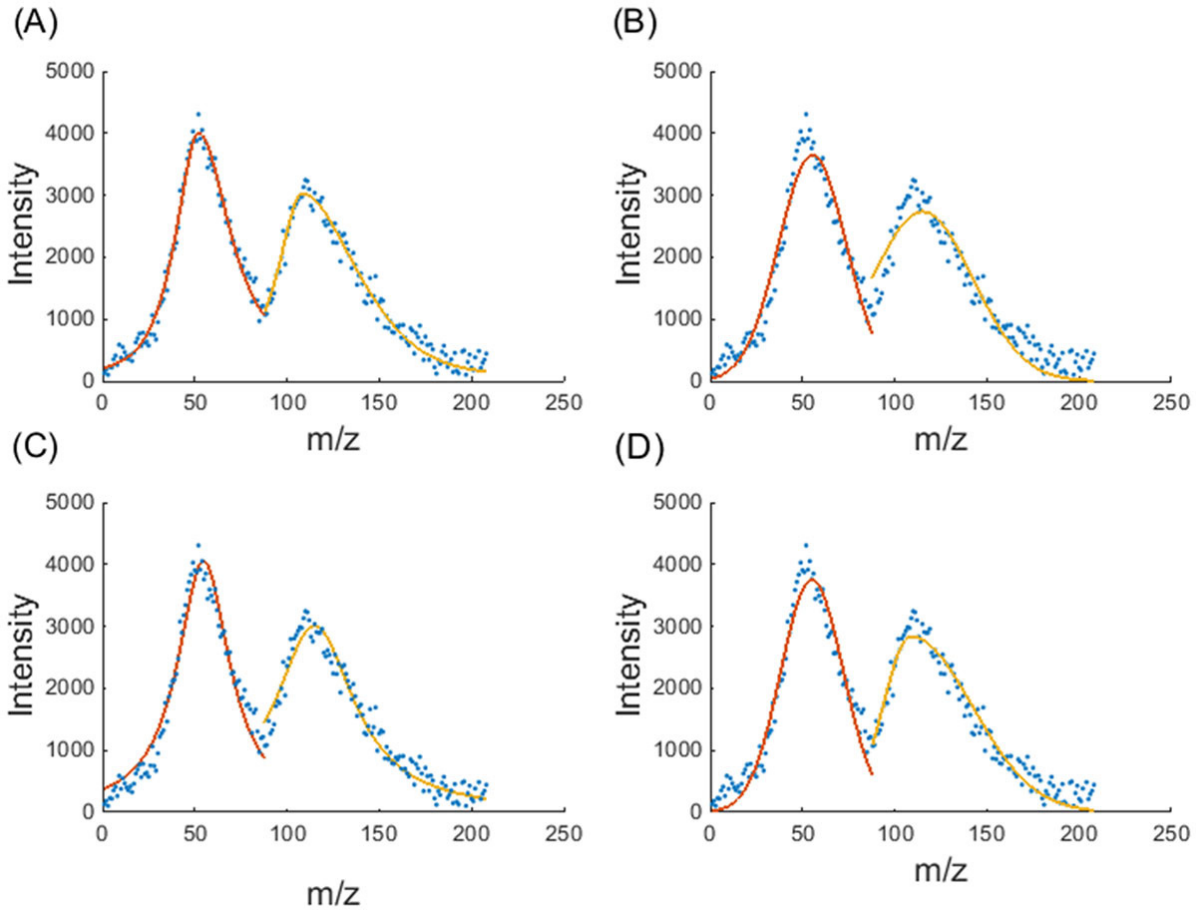
**Performance comparison of the peak models**. The peak fitting performance of (A) mAPV, (B) Gaussian, (C) Lorentz and (D) Bi-Gaussian models on two asymmetric and overlapped peaks is shown.

Figure 3 illustrates the peak fitting accuracies of the four models on two asymmetric and overlapped peaks. The proposed mAPV model delivered the best fit for both peaks compared to the other peak models. It can be clearly seen that the two symmetric peak models, Gaussian and Lorentz, have failed to provide a better fit to the two asymmetric peaks, and have estimated the peak summit locations less accurately. The Bi-Gaussian model appears to perform well for the second peak. However, in comparison to the proposed mAPV model, the Bi-Gaussian model has delivered a less accurate fit to the second half of that peak, which appears to follow the Lorentzian shape.

The precise estimation of peak summit location and peak area are of high importance in order to identify the corresponding bio-molecules and to determine their abundances accurately. Therefore, the above results validate the use of the mAPV model for MS peak modelling.

### Comparison of the performance of the proposed algorithm against other peak detection algorithms

Using the simulation datasets generated from the MALDI-TOF computer model, we performed a comparison between the proposed algorithm and two other widely used peak detection algorithms. The first benchmark algorithm uses CWT along with ridge lines for peak detection [10]. After a comprehensive study of various peak detection algorithms, Yang *et al*. demonstrated the advantages of using this method [9]. The second benchmark algorithm, which is known as Cromwell, is mainly based on UDWT and signal to noise ratio of peaks [11]. It also has been widely used in MS peak detection. Therefore, we selected these two algorithms as benchmarks in order to evaluate the performance of the proposed algorithm.

Well established performance measures such as sensitivity, FDR and F1-score were used to evaluate and compare the performance of this new pipeline. We applied all three algorithms to 2500 simulated spectra and calculated the above measures for each algorithm on each spectrum. The sensitivity is defined as the percentage of correctly identified peaks out of the total number of real peaks, whereas FDR is the percentage of incorrectly identified peaks out of the total number of identified peaks. The F1-score, which combines both sensitivity and FDR, is defined as follows [9]:

(7) $$F1-score=\frac{2\times \left(1-FDR\right)\times Sensitivity}{1-FDR+Sensitivity}.$$

In this comparison study, peaks that were located within ±1% error range of a known *m/z *value corresponding to a real peak were considered as true peaks. Moreover, both algorithms need some parameters to be set. In this study, the parameter values recommended by Yang *et al*. were used [9].

For these simulated spectra, Table 3 demonstrates that the performance of the proposed algorithm is better than the other benchmark methods in terms of sensitivity. It achieved a sensitivity of 85%, in comparison with 77% and 71% of CWT and Cromwell methods respectively. The utilization of an additional step to decompose broad and highly asymmetric peaks improved the sensitivity of the proposed algorithm. In particular, it facilitates the identification of most of the peaks corresponding to the observed isotopologue distribution for any particular bio-molecule. Isotopologues of an individual molecule vary only in terms of isotopic compositions. The peaks corresponding to isotopologues may have overlapping distributions to other molecules with very close mass causing the algorithm to detect a single highly asymmetric peak. Such peaks need to be decomposed accurately to identify all of the isotopologues and to provide accurate measurements of peak areas. If not, the peak detection algorithms with symmetric peak models would fail to identify any of the peaks in the overlapping distribution. Algorithms, which use asymmetric peak models, would detect only the highest abundant isotopologue, however, an inaccurate value would be given as its abundance.

**Table 3:** Performance of different peak detection algorithms in terms of sensitivity, FDR and F1-score.

Method	Sensitivity (%)	FDR (%)	F1-score (%)
CWT	76.74 ± 0.14	31.47 ± 0.36	70.77 ± 0.19
			
Cromwell	70.76 ± 0.13	49.92 ± 0.10	58.50 ± 0.09
			
New Algorithm	84.66 ± 0.10	32.70 ± 0.22	74.36 ± 0.15

Values are expressed as mean ± SE.

The problem of having overlapped peaks is quite common in data obtained through MS instruments with low resolving power. The modern instruments with high resolving power have the ability to distinguish all peaks in mass spectra from each other without generating overlapped peaks, thereby avoiding the need for peak decomposition [25]. However, the inability of most of the TOF MS instruments to attain this level of resolving power, calls for efficient peak detection algorithms that address this issue.

**Figure 4:**
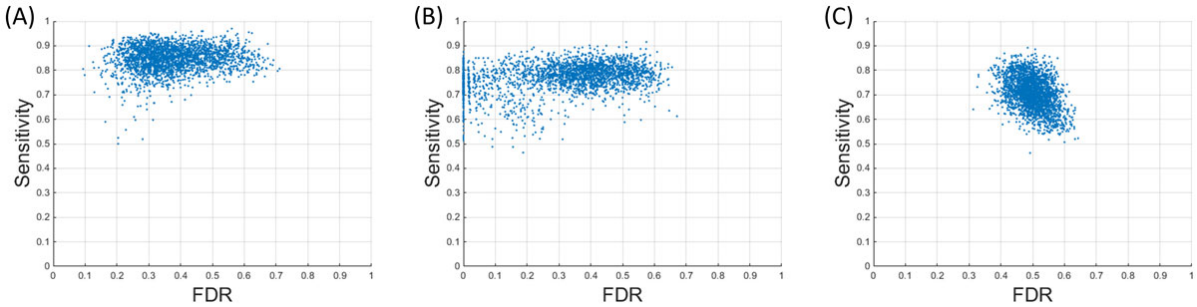
**Performance comparison of the peak detection algorithms**. Performance of (a) the proposed algorithm, (b) CWT based algorithm and (c) Cromwell algorithm on simulated MALDI-TOF-MS data is shown. Each scatter plot illustrates the Sensitivity against FDR.

When compared to the CWT method, a slight increase in FDR was observed in the proposed algorithm. Therefore, we used the F1-score, which combines both sensitivity and FDR, to compare the performances of the three algorithms. The proposed new algorithm delivered the highest F1-score of 74% compared to 71% and 58% of the CWT and Cromwell algorithms respectively. The 3.59% difference in F1-score between the proposed algorithm and its closest competitor - the CWT method, was found to be statistically significant through a t-test (p-value < 0.05). The scatter plots of sensitivity and FDR illustrate the complete behaviour of the three algorithms (Figure 4). The proposed algorithm appears to maintain a higher sensitivity in peak detection across all spectra. These results validate the use of the proposed peak detection algorithm in MALDI-MS data analysis.

Moreover, we observed that more than 42% of the false peaks detected by the proposed algorithm lie in the m/z range below 5000, which is the range affected by the baseline in this dataset. Therefore, we suggest improving the proposed algorithm by incorporating advanced baseline removal techniques in order to reduce the FDR. The proposed algorithm took about 8 minutes to detect peaks in a mass spectrum containing around 70 peaks on average, on a Windows 7 (64-bit) operating system running on a Core™i7-2600 CPU at 3.40GHz with 8.0GB Random Access Memory.

In order to reduce the computational overhead, some studies have proposed to incorporate the average spectrum of each dataset for peak detection [12,19]. Therefore, we further evaluated the performance of the peak detection algorithms using the average spectra corresponding to the 25 simulated datasets. For this comparison, we used the previously selected two benchmark algorithms as well as another two recently proposed Bayesian nonparametric models namely LARK-HP (Levy Adaptive Kernel Regression with highest posterior realization) and LARK-MA (Levy Adaptive Kernel Regression with local modes under model averaging) proposed by House *et al*. for peak detection in MALDI-TOF-MS data [19]. These models need many parameters to be set. In this study, the parameter values recommended by House *et al*. were used and both algorithms were run for one hundred thousand iterations on each average spectrum [19].

Table 4 summarizes the peak detection performance of the proposed algorithm and four benchmark algorithms on average spectra. The average sensitivities of the proposed algorithm and the CWT method were decreased with the use of average spectra. However, the FDRs of these two algorithms were also reduced by 18.61% and 29.80% respectively. Although the Cromwell algorithm obtained a higher average sensitivity of 74% on average spectra, its FDR (38.76%) is 18.01% to 37.09% higher than that of the other algorithms. LARK-HP and LARK-MA methods attained lower FDRs when compared to the Cromwell algorithm. However, these two Bayesian nonparametric models failed to outperform the other algorithms in terms of sensitivity. These results suggest applying the proposed algorithm and the CWT method on individual spectra, to achieve better peak detection results in terms of sensitivity. However, in order to obtain a lower FDR, these algorithms, particularly the CWT method, can be applied on average spectra.

**Table 4:** Performance of different peak detection algorithms on average spectra in terms of sensitivity, FDR and F1-score.

Method	Sensitivity (%)	FDR (%)	F1-score (%)
CWT	65.84 ± 0.64	1.67 ± 0.45	78.81 ± 0.44
Cromwell	74.40 ± 0.99	38.76 ± 0.59	67.11 ± 0.62
LARK-HP	65.73 ± 0.85	20.75 ± 1.72	71.58 ± 0.85
LARK-MA	56.16 ± 0.57	17.65 ± 1.75	66.59 ± 0.75
New Algorithm	68.96 ± 0.72	14.09 ± 0.47	76.46 ± 0.54

Values are expressed as mean ± SE.

### Dual-tree complex wavelet transformation (DT-CWT) for spectra smoothing

**Figure 5:**
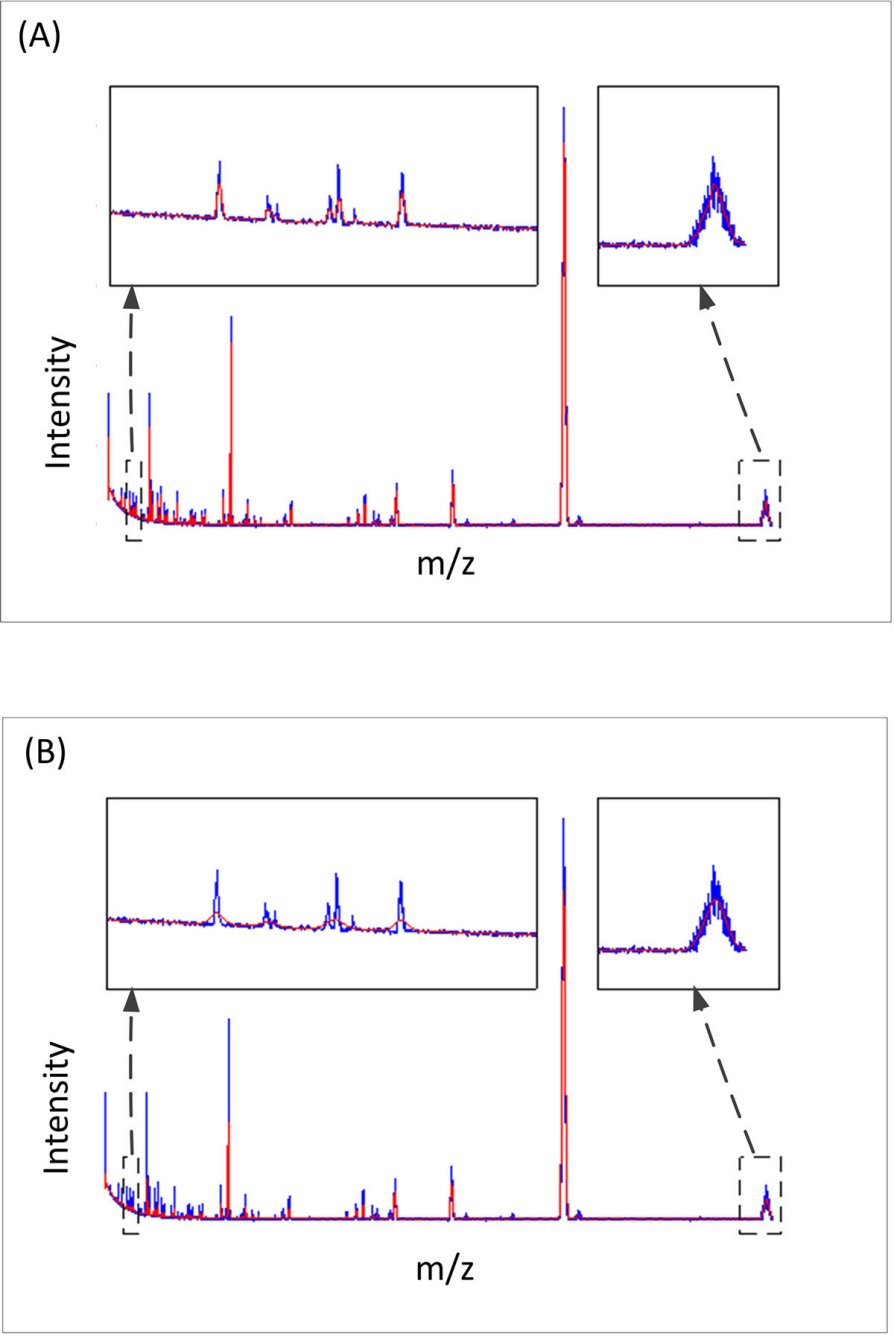
**The effect of threshold value on spectra smoothing**. A raw spectrum (blue) with the corresponding smoothed spectra (red) obtained using (A) a smaller threshold value and (B) a larger threshold value are shown. When a smaller threshold value is used, peaks at higher masses appear to be under-smoothed whereas when a larger threshold value is used, peaks at lower masses appear to be over-smoothed.

**Figure 6:**
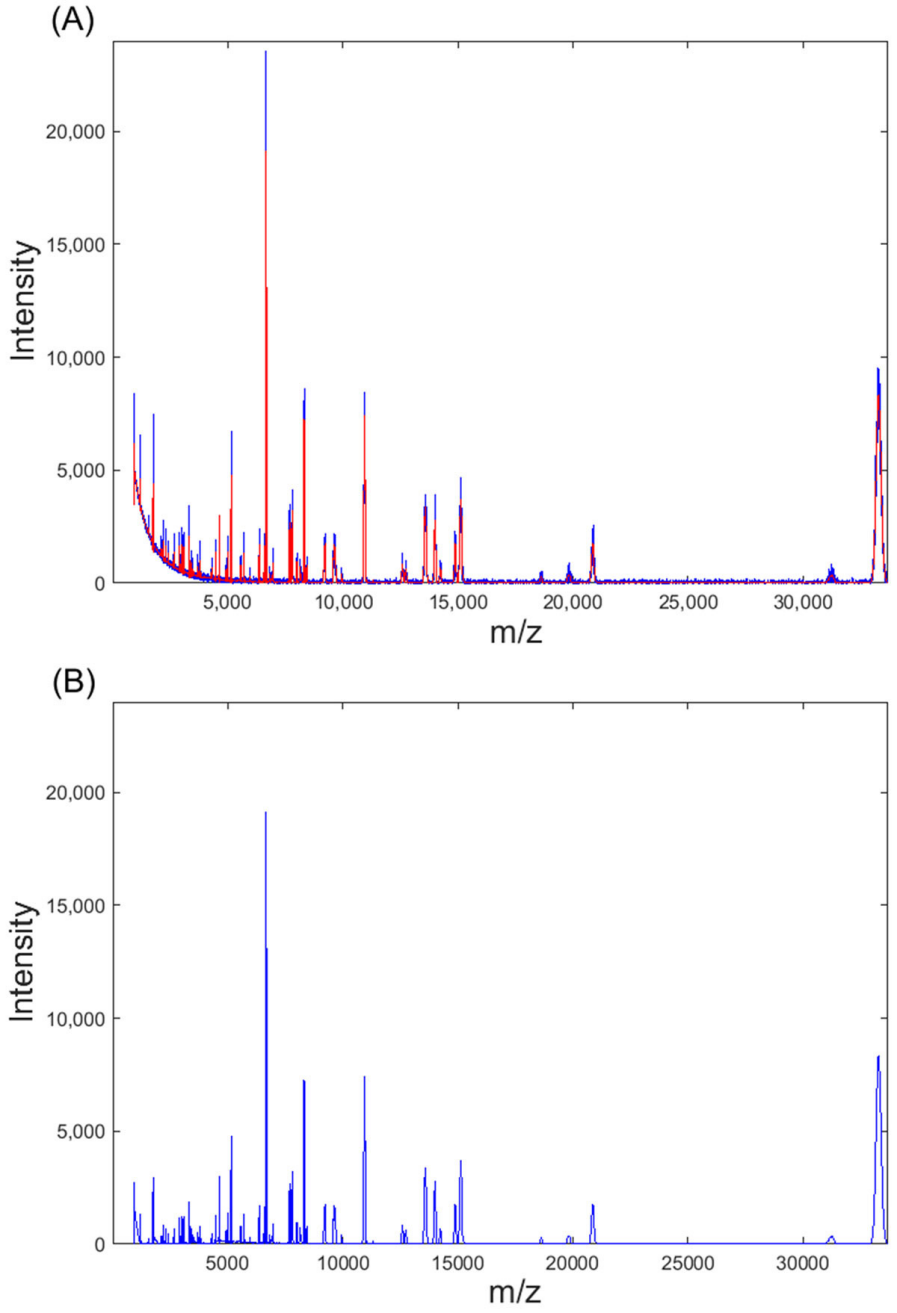
**An example of spectra pre-processing**. This figure shows (A) a raw spectrum (blue) along with the spectrum after smoothing (red) and (B) the spectrum after baseline correction.

As the threshold value at each level in the wavelet domain is determined using SURE, the proposed spectra smoothing method does not require any parameter to be set by the user, thereby avoiding the additional overhead of customizing the method for different datasets. Moreover, the noise in a mass spectrum decreases along the spectrum. Hence the use of a global threshold value calculated using the entire spectrum can result in either over-smoothing or under-smoothing. Figure 5 shows smoothing results obtained using two different threshold values. The smaller threshold value works well for the peaks at lower masses. However, it tends to under-smooth the peaks at higher masses. Conversely, the larger threshold value yields better smoothing results for the peaks at higher masses while over-smoothing the peaks at lower masses. This problem could be reduced by dividing each spectrum into 4 equal segments and calculating the threshold values for each segment separately. Figure 6 shows a spectrum smoothed using this method along with the corresponding baseline-corrected spectrum.

### The modified Asymmetric Pseudo-Voigt (mAPV) function for modelling MS peaks

Symmetric Gaussian and Lorentz functions have been used in the context of MS peak modelling. The Voigt function combines both Gaussian and Lorentz functions using convolution operation. Hence, it can be used to get the best from both these models. Pseudo-Voigt function, which is a close approximation to the conventional Voigt function, uses a linear combination of Gaussian and Lorentz functions instead of convolution in order to reduce the computational overhead.

**Figure 7:**
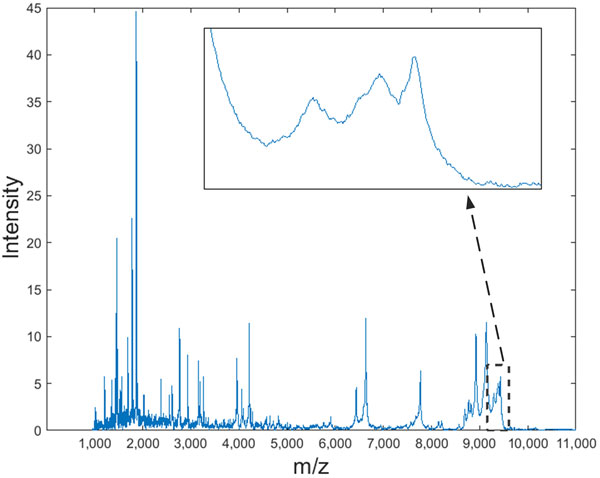
**A real MALDI-TOF mass spectrum containing asymmetric and overlapping peaks**. The zoomed region shows asymmetric and overlapping peaks in the higher mass range. This spectrum was extracted from the dataset provided in Alexandrov *et al*. [1].

Moreover, most of the existing studies use symmetric peak models assuming all the peaks in mass spectra to be symmetric. However, asymmetric peaks can also be observed in mass spectra [10]. Figure 7 shows an example of a real MALDI-TOF mass spectrum containing asymmetric and overlapping peaks. This spectrum, which represents the serum protein profile of a colorectal cancer patient, was extracted from a dataset provided in Alexandrov *et al*. [1]. The symmetric peak models produce inaccurate estimations for peak parameters of these asymmetric peaks.

**Figure 8:**
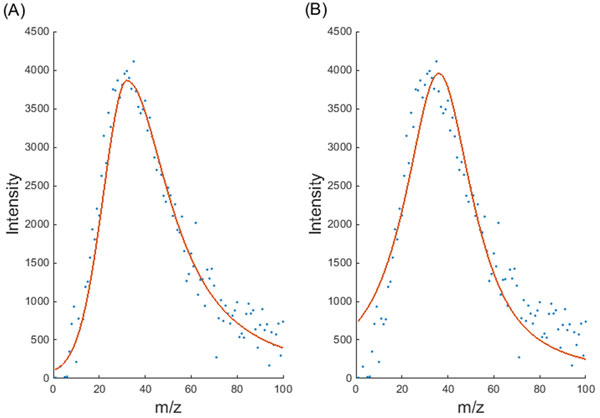
**Performance comparison of the mAPV and the standard APV models**. The peak fitting performance of (A) mAPV and (B) standard APV models on an asymmetric peak, having dissimilar proportions of Gaussian and Lorentz functions for the two halves of the peak, is shown.

The mAPV model proposed in this paper has a characteristic shape (Figure 1) and it is not a black box model where there is no physical meaning to the parameters. There is an important application specific reason behind the introduction of each parameter to this model. For example, σ_1 _and σ_2 _were used instead of one σ parameter in order to account for asymmetric peaks and β_1 _and β_2 _parameters were introduced to the model to provide a better fit for peaks with different shapes. The proposed mAPV model is different to the APV function used in the literature because unlike the latter the former can have different proportions of the Lorentz and Gaussian functions for the two halves of the peak. Sometimes in mass spectra, peaks can be found in which one half of the peak can be modelled accurately using the Gaussian function and the other half of the peak can be accurately modelled using the Lorentz function. For such peaks, the mAPV function provides a better fit as it can represent both Gaussian and Lorentz functions individually as well as various combinations of them. Figure 8 shows an asymmetric peak in which the first half follows the Gaussian shape and the second half follows the Lorentzian shape. It can be clearly seen that the APV function has failed to provide an adequate fit for this peak, as it assumes the proportions of the Gaussian and Lorentz functions to be same for both halves of the peak. In contrast, the additional parameter has allowed the mAPV model to provide a better fit for this asymmetric peak.

## Conclusions

This paper presents a new peak detection and parameter estimation algorithm for MALDI-MS data. It incorporates DT-CWT along with SURE for spectra smoothing thus avoiding the overhead of customizing the method for different datasets by setting parameters. It also proposes the mAPV model to fit MS peaks more accurately.

We have successfully applied the proposed unsupervised algorithm on MS data, generated from a MALDI-TOF computer model, to detect peaks and to estimate the peak parameters. In particular, it has been demonstrated that the proposed mAPV model with an optimization algorithm is a promising method to model peaks in MS data, which aids in identifying underlying bio-molecules and determining their abundances accurately. The proposed algorithm has a potential advantage over the existing methods for low resolution MS data having overlapped peak distributions and asymmetric peaks, which is quite common in metabolomics and proteomics studies.

We believe that these results can be further improved by incorporating the available important details regarding the dataset under study. Although we propose this algorithm for peak detection in MALDI-MS data, we believe that it can also be used for data generated by other types of MS instruments such as LC-MS and SELDI-MS. We also suggest improving the proposed algorithm to be used for peak detection in MALDI-IMS data, incorporating the additional information about the spatial distribution of bio-molecules generated by this technology.

## Appendix

### Appendix 1: Stein's Unbiased Risk Estimator (SURE)

Let $\mu =\left(\mu_{1},\mu_{2},\ldots ,\mu_{d}\right)$ be a parameter vector and $x=\left(x_{1},x_{2},\ldots ,x_{d}\right)$ be a measurement vector where$x_{i}\sim N\left(\mu_{i},\sigma^{2}\right)$. If *h*(*x*) is an estimator of *μ *such that$h\left(x\right)=x+g\left(x\right)$, $g={\left(g_{i}\right)}_{i=1}^{d}$ and *g*(*x*) is weakly differentiable, SURE is defined as follows [21]:

$$SURE\left(h\right)=d\sigma^{2}+||g{\left(x\right)}^{2}||+2\sigma^{2}\sum_{i=1}^{d}\frac{\partial }{\partial x_{i}}g_{i}\left(x\right),$$

where ||·|| is the Euclidean norm.

### Appendix 2: Pseudocode of the Hierarchical Particle Swarm Optimization (HPSO) algorithm

Let *n *be the number of particles in the swarm and *d *be the dimensionality of the search space. Moreover, suppose *Xi *= (*x*_*i*1_, *x*_*i*2_,... *x*_*id*_) is the position of the *i*^th ^particle, *Vi *= (*v*_*i*1_, *v*_*i*2_,..., *v*_*id*_) is its velocity vector, *Pi *= (*p*_*i*1_, *p*_*i*2_,..., *p*_*id*_) is its personal best position and *P_g _*= (*p*_*g*1_, *p*_*g*2_,..., *p*_*gd*_) is the best particle found so far. The pseudocode of the HPSO algorithm is as follows [24]:

Begin

   Initialize the population

   **while **(termination condition = false) **do**

      **for **(*i *= 1 to *n*)

         Evaluate fitness

         Update *p_id_*

         Update *p_gd_*

         **for **(*d *= 1 to dimensionality of the search space)

            Calculate *v_id_*

            Update *x_id_*

         **end for**

      **end for**

   **end while**

**End**.

### Appendix 3: Gaussian, Lorentz and Bi-Gaussian functions

#### Gaussian function

Gaussian function has 3 parameters namely height of the peak (*H*), location of the peak summit (*α*) and standard deviation of the peak (*σ*). When this function is used to model peaks in mass spectra, the intensity values of a peak can be modelled as a function of mass values (*m*) as follows:

$$G\left(m\right)=H\times e^{\left[-ln\left(2\right){\left(\frac{m-\alpha }{\sigma }\right)}^{2}\right]}.$$

#### Lorentz function

Lorentz function has 3 parameters namely height of the peak (*H*), location of the peak summit (*α*) and standard deviation of the peak (*σ*). When this function is used to model peaks in mass spectra, the intensity values of a peak can be modelled as a function of mass values (*m*) as follows:

$$L\left(m\right)=H\times \left[\frac{1}{1+{\left(\frac{m-\alpha }{\sigma }\right)}^{2}}\right].$$

#### Bi-Gaussian function

Bi-Gaussian function has 4 parameters namely height of the peak (*H*), location of the peak summit (*α*), standard deviation of the first half of the peak (*σ*_1_) and that of the second half of the peak (*σ*_2_). When this function is used to model peaks in mass spectra, the intensity values of a peak can be modelled as a function of mass values (*m*) as follows:

$$B\left(m\right)=\left\{\begin{array}{c} H\times e^{\left[-ln\left(2\right){\left(\frac{m-\alpha }{\sigma_{1}}\right)}^{2}\right];m<\alpha }\\ H\times e^{\left[-\text{ln}\left(2\right){\left(\frac{m-\alpha }{\sigma_{2}}\right)}^{2}\right];m\geq \alpha } \end{array}\right).$$

## Competing interests

The authors declare that they have no competing interests.

## Authors' contributions

CDW developed the methods, assessed the performance and drafted the manuscript. BAB and UR offered the knowledge in mass spectrometry based biological research and contributed to design the study. IS and SKH provided assistance to improve the developed methods. All authors edited and approved the final manuscript.

## Supplementary Material

Additional file 1 **Supplementary Information**. This file contains detailed information about the simulation dataset used to evaluate the proposed mAPV peak model. Click here for file

## Declarations

Publication charges for this article have been jointly funded by The University of Melbourne and Metabolomics Australia.

This article has been published as part of *BMC Genomics *Volume 16 Supplement 12, 2015: Joint 26th Genome Informatics Workshop and 14th International Conference on Bioinformatics: Genomics. The full contents of the supplement are available online at http://www.biomedcentral.com/bmcgenomics/supplements/16/S12.
