# Supplementary material for: A new peak detection algorithm for MALDI mass spectrometry data based on a modified Asymmetric Pseudo-Voigt model
Source: BMC Genomics. 2015 Dec 9;16(Suppl 12):S12. doi: 10.1186/1471-2164-16-S12-S12 (PMC4682410; doi:10.1186/1471-2164-16-S12-S12)
Supplement: Additional file 1 — Supplementary Information. This file contains detailed information about the simulation dataset used to evaluate the proposed mAPV peak model. [file 1471-2164-16-S12-S12-S1.pdf]

Additional file 1

Simulation data to evaluate the mAPV model

Table S1 contains the 12 parameters and their values used to generate the first simulation dataset. In order to assess the peak fitting accuracy for different levels of peak asymmetry and peak overlap, 2700 parameter combinations were chosen by varying 7 parameters ($\sigma_{1,2}$, $\sigma_{2,2}$, $\beta_{1,1}$, $\beta_{1,2}$, $\beta_{2,1}$, $\beta_{2,2}$ and α_2). Each parameter combination was tested 100 times.

Table S1 – Parameter values used to generate the first simulation dataset.

Parameter	Description	Value/s
α_1	Summit location of the first peak	50
H_1	Height of the first peak	4000
$\sigma_{1,1}$	Standard deviation of the first half of the first peak	12
$\sigma_{1,2}$	Standard deviation of the second half of the first peak	$\sigma_{1,1} \times$ a ; a = 1, 1.25, 1.5, 1.75, 2
$\beta_{1,1}$	The fraction of Lorentz function in the first half of the first peak	0, 0.5, 1
$\beta_{1,2}$	The fraction of Lorentz function in the second half of the first peak	0, 0.5, 1
α_2	Summit location of the second peak	$\alpha_1 + (\sigma_{1,2} + \sigma_{2,1})b$; b = 1.5, 2, 4
H_2	Height of the second peak	3000
$\sigma_{2,1}$	Standard deviation of the first half of the second peak	16
$\sigma_{2,2}$	Standard deviation of the second half of the second peak	$\sigma_{2,1} \times$ c ; c = 1, 1.25, 1.5, 1.75, 2
$\beta_{2,1}$	The fraction of Lorentz function in the first half of the second peak	0.25, 0.75
$\beta_{2,2}$	The fraction of Lorentz function in the second half of the second peak	0.25, 0.75
